# Transcranial magnetic stimulation-based evaluation of exercise training-induced changes in TMS-derived neurophysiological markers and motor performance in healthy adults: a systematic review and meta-analysis of RCTs

**DOI:** 10.3389/fneur.2026.1832760

**Published:** 2026-06-25

**Authors:** Yili Gao, Ziwen Zhen, Juanjuan Hu

**Affiliations:** 1 College of Physical Education, Zhengzhou University of Technology, Zhengzhou, Henan, China; 2 Sport Innovation & Technology Center, Institute of Human-Centered Engineering, Universiti Teknologi Malaysia, Johor Bahru, Malaysia; 3 Department of Foundational Education, Sichuan Film and Television University, Chengdu, Sichuan, China; 4 Department of Physical Education, Chengdu College, University of Electronic Science and Technology of China, Chengdu, China

**Keywords:** corticospinal excitability, exercise training intervention, meta-analysis, motor performance, motor training, randomized controlled trials, TMS-derived neurophysiological markers, transcranial magnetic stimulation (TMS)

## Abstract

**Background:**

Transcranial magnetic stimulation (TMS) is a well-established, non-invasive technique for assessing motor cortical and corticospinal function, including corticospinal excitability and intracortical inhibition/facilitation. Because commonly used TMS outcomes reflect different neurophysiological mechanisms, they should not be interpreted as a single construct of “cortical plasticity.” The effects of structured exercise training on these TMS-derived markers and motor performance in healthy adults remain to be systematically clarified.

**Objective:**

This systematic review and meta-analysis synthesized evidence from randomized controlled trials (RCTs) to evaluate the effects of exercise training interventions on TMS-derived neurophysiological markers and motor performance in healthy adults.

**Methods:**

Following PRISMA guidelines, we conducted a comprehensive search of PubMed, Web of Science, Embase, and the Cochrane Central Register of Controlled Trials from inception to December 31, 2025. We included RCTs involving healthy adults (≥18 years) that compared structured exercise training interventions (e.g., resistance, aerobic, skill, balance, or combined training) with control conditions and used TMS to assess neurophysiological outcomes. Data were pooled using random-effects meta-analyses to calculate standardized mean differences (Hedges' g). Heterogeneity, exploratory subgroup differences, sensitivity, and publication bias were examined.

**Results:**

Twelve randomized controlled trials met the inclusion criteria. Across these studies, 14 effect sizes were available for TMS-derived neurophysiological markers and 16 effect sizes were available for motor-performance outcomes. The corresponding analytic samples were 137 exercise and 136 control participants for TMS-derived markers, and 154 exercise and 152 control participants for motor performance. Random-effects meta-analysis showed statistically significant pooled effects for TMS-derived neurophysiological markers (Hedges' g = 0.53; 95% CI: 0.10 to 0.95; *p* < 0.05) and motor performance (Hedges' g = 0.58; 95% CI: 0.23 to 0.93; *p* < 0.01). Moderate heterogeneity was observed for both outcomes (I^2^ = 64.8% and 54.0%, respectively), and prediction intervals crossed zero. Exploratory subgroup analyses suggested possible differences by intervention duration and training modality, but the duration meta-regression was non-significant and several subgroups had very small K. These subgroup findings should therefore be interpreted as hypothesis-generating rather than confirmatory. Certainty of evidence, assessed via GRADE, was moderate for both outcomes after downgrading for risk of bias, inconsistency, and possible publication bias.

**Conclusion:**

Exercise training interventions may improve motor performance and modulate TMS-derived neurophysiological markers in healthy adults, but the certainty and generalizability of these findings are limited. The heterogeneity of TMS markers, moderate between-study heterogeneity, prediction intervals crossing zero, small subgroup sizes, low statistical power of several trials, and possible small-study effects mean that the pooled results should not be interpreted as uniform or definitive effects. Current evidence is insufficient to establish the superiority of any specific training duration or modality. More standardized, adequately powered RCTs are needed to confirm prescription parameters and long-term effects.

**Systematic review registration:**

https://www.crd.york.ac.uk/prospero/display_record.php?ID=CRD420261343170, identifier: CRD420261343170.

## 1 Introduction

Transcranial Magnetic Stimulation (TMS), first introduced by Barker et al. in 1985, has become a key technique in human motor neuroscience (1). TMS applies brief magnetic pulses to the scalp to elicit neural responses from the motor cortex and corticospinal pathway. It allows quantification of TMS-derived neurophysiological markers such as motor evoked potential (MEP) amplitude, resting motor threshold (RMT), short-interval intracortical inhibition (SICI), and intracortical facilitation (ICF) (2–4). These measures provide complementary, rather than interchangeable, information about corticospinal excitability, intracortical inhibition/facilitation, and motor cortical function. Therefore, in the present review, these outcomes are described as TMS-derived neurophysiological markers rather than as a single construct of cortical plasticity.

Exercise training interventions are widely known to improve motor performance and may also influence central motor pathways. Different types of training are likely to affect TMS-derived markers in different ways. Resistance training can increase neural drive and optimize motor-unit recruitment, which may be reflected in corticospinal responses (5, 6). Motor skill training can improve coordination and precision by repeatedly engaging task-specific sensorimotor circuits (7). High-intensity interval training and combined training may also influence cortical and corticospinal function through repeated activation, sensory feedback, and fatigue-resistance demands (8, 9). These adaptations may involve changes in both excitatory and inhibitory processes, and the balance between them is important for motor function (10).

Despite extensive research on exercise, cortical excitability, and motor performance, important gaps remain. First, the effects of different exercise-training modalities on TMS-derived neurophysiological markers have not been systematically compared in healthy adults (11). Second, substantial heterogeneity exists across studies because of differences in participant characteristics, training protocols, target muscles, and TMS outcomes. This heterogeneity limits the stability and mechanistic interpretation of pooled estimates. Third, many trials in this field have small sample sizes, which increases imprecision and the possibility of small-study effects (12, 13).

Given these challenges, this study aimed to synthesize RCT evidence on the effects of exercise training interventions on TMS-derived neurophysiological markers and motor performance in healthy adults. We also aimed to evaluate heterogeneity, explore potential moderators, and interpret the findings cautiously in light of marker-specific mechanisms and study-level limitations.

Scope statement: The review is positioned within human motor and systems neuroscience, focusing on how structured exercise training relates to TMS-derived motor cortical and corticospinal markers and behavioral motor outcomes.

## 2 Study methods

### 2.1 Inclusion and exclusion criteria

Eligibility criteria were defined according to the PICOST framework, covering the following domains: study design, participants, interventions, comparators, outcomes, and timing. Study selection was conducted independently by two reviewers. Any disagreements were resolved through discussion, and when necessary, a third reviewer was consulted to reach a final decision. The review protocol was prospectively registered in PROSPERO (CRD420261343170).

Terminology: Throughout this review, “exercise training intervention” refers to a planned and structured program of repeated exercise sessions with a defined modality, frequency, intensity, and duration. This term replaces the less precise wording “periodic exercise.”

#### 2.1.1 Inclusion criteria

(1) The study subjects were healthy adults aged ≥18 years, with no history of neurological, musculoskeletal, or psychiatric disease; no restrictions were imposed on gender or training background. (2) The study design was a randomized controlled trial (RCT). (3) The intervention consisted of a structured exercise training intervention, including but not limited to resistance training, aerobic training, skill training, balance training, and high-intensity interval training. (4) The study used TMS to evaluate TMS-derived neurophysiological markers of motor cortical or corticospinal function. (5) Outcome indicators included at least one TMS-derived neurophysiological marker or motor-performance measure. TMS-derived markers included MEP amplitude, RMT, SICI, ICF, and related measures; motor performance outcomes included muscle strength, power, endurance, balance, and other task-performance measures. (6) The literature provided extractable quantitative data.

#### 2.1.2 Exclusion criteria

(1) The study subjects included patients, rehabilitated individuals, athletes, minors, or animals. (2) The intervention was not exercise training, or the exercise intervention was insufficiently described. (3) The study did not use TMS to evaluate neurophysiological markers or did not report quantitative results related to TMS-derived markers or motor performance. (4) The study was not a randomized controlled trial, such as a cross-sectional study, cohort study, or case-control study. (5) The publication was a review, conference abstract, commentary, case report, duplicate publication, or a study for which the full text could not be obtained.

### 2.2 Literature search strategy

A systematic search of PubMed, Web of Science, Embase, and the Cochrane Central Register of Controlled Trials (CENTRAL) was conducted to identify relevant studies published up to December 31, 2025. The search strategy combined Medical Subject Headings (MeSH) and free-text terms and was constructed around four domains: population, intervention, assessment method, and outcomes. Population-related terms included “healthy adults,” “healthy individuals,” and “healthy subjects.” Intervention-related terms included “exercise intervention,” “exercise training,” “structured exercise training,” “resistance training,” “strength training,” “aerobic training,” “motor skill training,” and “high-intensity interval training.” Assessment-related terms included “transcranial magnetic stimulation,” “TMS,” “single-pulse TMS,” and “paired-pulse TMS.” Outcome-related terms included “TMS-derived neurophysiological markers,” “cortical excitability,” “corticospinal excitability,” “motor evoked potential,” “resting motor threshold,” “short-interval intracortical inhibition,” “intracortical facilitation,” “motor performance,” “muscle strength,” “endurance,” and “balance.” The search strategy was adapted for each database according to its specific retrieval requirements, and the Boolean operators “AND” and “OR” were used to combine search terms. The PubMed search strategy was as follows: (“Healthy Volunteers”[Mesh] OR “healthy adults” OR “healthy individuals” OR “healthy subjects”) AND (“Exercise”[Mesh] OR “Exercise Therapy”[Mesh] OR “exercise training” OR “physical training” OR “exercise intervention” OR “resistance training” OR “strength training” OR “aerobic training” OR “motor skill training” OR “high-intensity interval training” OR HIIT) AND (“Transcranial Magnetic Stimulation”[Mesh] OR “transcranial magnetic stimulation” OR TMS OR “single-pulse TMS” OR “paired-pulse TMS”) AND (“cortical plasticity” OR “cortical excitability” OR “corticospinal excitability” OR “motor evoked potential” OR MEP OR “resting motor threshold” OR RMT OR “short-interval intracortical inhibition” OR SICI OR “intracortical facilitation” OR ICF OR “motor performance” OR “muscle strength” OR endurance OR balance OR “physical performance”).

### 2.3 Literature screening and data extraction

#### 2.3.1 Literature screening process

EndNote and Zotero were used for literature import and duplicate removal. Two reviewers independently conducted the initial screening and subsequent full-text review. During initial screening, studies that were clearly irrelevant to the inclusion criteria were excluded based on title and abstract. Full-text articles were then reviewed to determine final inclusion according to the eligibility criteria. Disagreements were resolved through discussion, and a third reviewer was consulted when necessary. The literature screening process is presented using a corrected PRISMA 2020 flow diagram (14).

#### 2.3.2 Data extraction

Data extraction was performed independently by two researchers using a standardized form. Extracted data included corresponding author, publication year, journal, participant characteristics of experimental and control groups (e.g., age and sex), intervention type, training protocol, comparator, and outcome measures. For each outcome, post-intervention means, standard deviations, and sample sizes were extracted when available. When data were not reported in the text, authors were contacted first; if necessary, graphical data were extracted using appropriate software.

### 2.4 Bias risk assessment

The Cochrane risk-of-bias tool was used to evaluate the risk of bias. The included studies were assessed across five domains (D1–D5), and each domain was rated as Low risk, Some concerns, or High risk. An overall risk-of-bias judgement was assigned to each study according to the combined assessment across domains. These judgements were used to inform the interpretation of the findings and the GRADE assessment.

### 2.5 Statistical analysis

Meta-analysis was performed using R software. Analyses included effect-size aggregation, heterogeneity testing, forest plots, sensitivity analyses, meta-regression where feasible, and publication-bias assessment. Outcomes were continuous and measured with different units; therefore, standardized mean differences corrected for small-sample bias (Hedges' g) and 95% confidence intervals (95% CIs) were calculated. Because the included TMS outcomes reflected different physiological mechanisms, the pooled neurophysiological analysis was labeled as an aggregate of TMS-derived neurophysiological markers rather than as a single cortical-plasticity construct. Separate metric-specific meta-analyses were considered, but the number of effect sizes for individual TMS markers was insufficient for stable confirmatory estimates. Therefore, all subgroup and moderator analyses were treated as exploratory, and subgroups with very small K were interpreted as hypothesis-generating only. A random-effects model was prioritized because of expected clinical and methodological heterogeneity. Statistical significance was set at *P* < 0.05. Heterogeneity was evaluated with I^2^, where I^2^ <50% indicated low heterogeneity, 50% ≤ I^2^ <75% indicated moderate heterogeneity, and I^2^ ≥ 75% indicated high heterogeneity. Exploratory subgroup analyses included intervention duration (≥4 weeks vs. <4 weeks), training frequency ( ≤ 3 sessions/week vs. >3 sessions/week), and exercise type (strength, skill, and combined training). Funnel plots and Egger's test were used to assess possible publication bias and small-study effects when feasible.

### 2.6 Evidence certainty assessment

The GRADE (Grading of Recommendations Assessment, Development and Evaluation) approach was used to rate the certainty of evidence for the primary outcomes, considering risk of bias, inconsistency, indirectness, imprecision, and publication bias. The certainty of evidence was categorized as high, moderate, low, or very low (15).

## 3 Results

### 3.1 Search results

**Figure 1:**
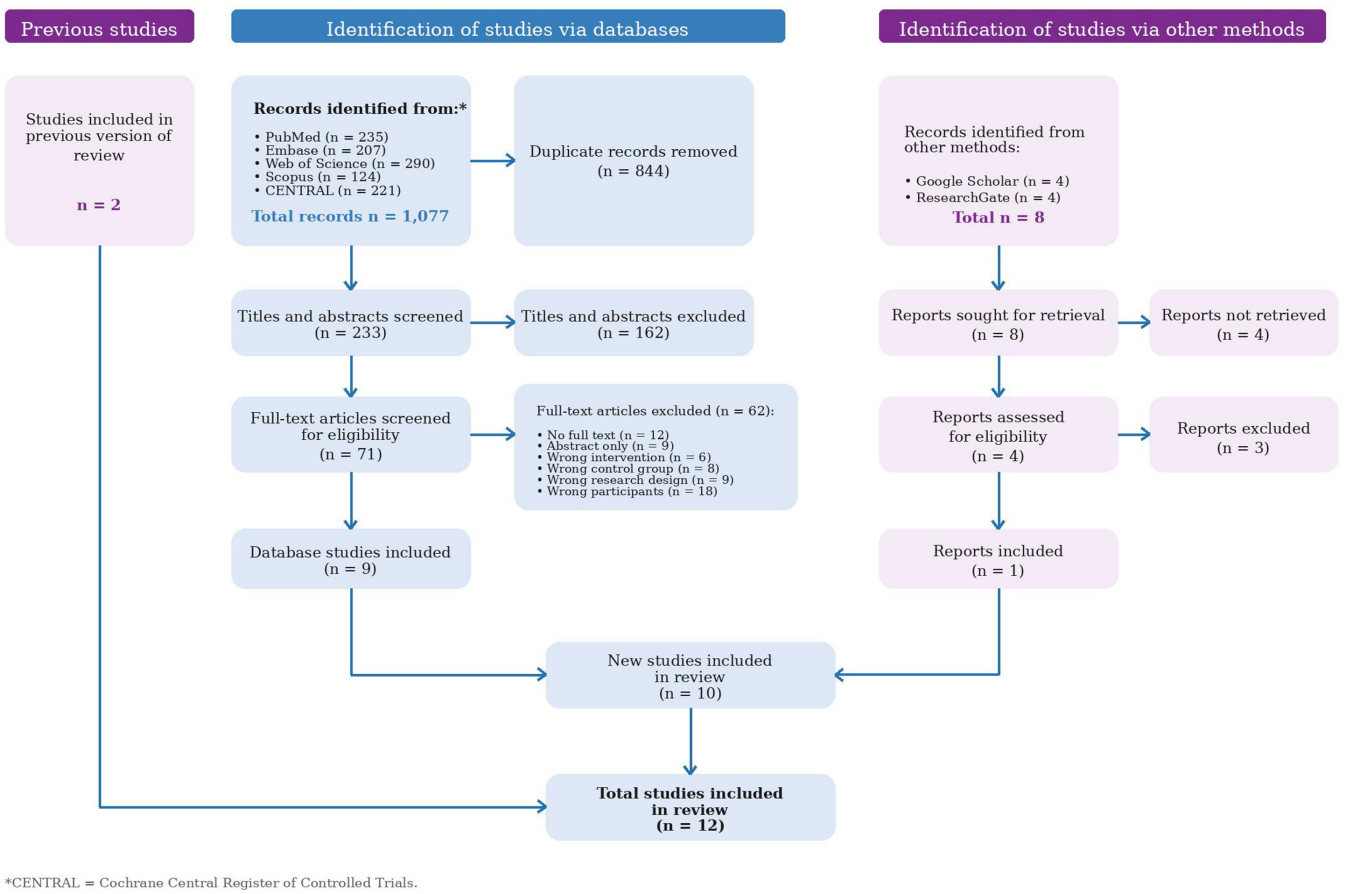
Corrected PRISMA 2020 flow diagram of literature search and study selection.

Database searches identified 1,077 records. After 844 duplicates were removed, 233 records remained for title and abstract screening, of which 162 were excluded. Seventy-one database records underwent full-text screening; 62 were excluded for reasons shown in Figure 1. Searches by other methods identified eight additional reports; four were retrieved and assessed, and three were excluded. Ten new studies were included and, together with two studies identified from the previous review version, 12 studies were included in the final review. The corrected PRISMA 2020 flow diagram is shown in Figure 1.

### 3.2 Basic Characteristics of the included studies

A total of 12 randomized controlled trials were included in this review, published between 2005 and 2021, all involving healthy adults. The sample sizes of the included studies were generally small, with 7–33 participants in the intervention groups and 7–12 participants in the control groups. Most studies included both male and female participants. The interventions mainly consisted of resistance training, motor skill training, balance training, and combined training, with resistance training being the most common. The trained muscle groups primarily included the wrist flexors, wrist extensors, biceps brachii, first dorsal interosseous muscle, and lower-limb muscle groups. Control conditions commonly included no training, maintenance of usual daily activities, seated rest, non-progressive skill training, or performing the same movements without external loading. The intervention duration ranged from a single session to 6 weeks, with 4 weeks being the most common duration. Training frequency was most commonly three sessions per week. Outcome measures fell into two categories: motor performance outcomes and TMS-derived neurophysiological markers. Motor performance outcomes included one-repetition maximum (1RM), maximal voluntary contraction (MVC), dynamic strength, motor performance scores, balance performance, and voluntary activation. TMS-derived markers included MEP amplitude, maximal MEP, motor threshold, and SICI. These markers were not assumed to represent one identical physiological mechanism. Overall, considerable heterogeneity was present in intervention type and outcome measures, providing a basis for cautious exploratory subgroup analyses (Table 1).

**Table 1:** Basic characteristics of the included studies.

Study	Study design	Male/ female	Sample size	Experimental intervention	Control intervention	Duration (weeks)	Frequency (sessions/week)	Outcome measures
Christiansen et al. (38)	RCT	24/0	12/11	Progressive motor skill training	Non-progressive skill training	6	3	Exercise performance score, motor threshold
Mason et al. (39)	RCT	10/8	9/9	High-intensity wrist flexion (right hand, 80% 1RM)	Seated rest 15 min	2	3	One-repetition maximum strength increase, short-interval cortical inhibition
Coomb et al. (40)	RCT	11/12	16/7	Wrist extensor dynamic strength training (dumbbells)	No training, maintain daily activity	3	3	One-repetition maximum dynamic strength (wrist extensors), motor evoked potential amplitude
Jensen et al. (25)	RCT	13/11	16/8	Strength training (right biceps) + motor skill training (right elbow flexion skill)	No training, participate in all tests	4	3	Average maximal dynamic muscle strength (1RM), maximal motor evoked potential
Kidgell et al. (26)	RCT	13/3	8/8	Index finger abduction	No training	4	2.5	Maximal voluntary contraction, motor evoked potential amplitude
Nuzzo et al. (27)	RCT	12/9	10/11	12 high-intensity isometric elbow flexions	Sitting, same duration	4	3	Maximal strength, voluntary activation
Siddique et al. (28)	RCT	22/20	32/10	Progressive/velocity isometric strength training	No training	4	3	One-repetition maximum (kg, dynamic elbow flexion), short-interval cortical inhibition
Carroll et al. (29)	RCT	11/6	8/9	Right wrist radial deviation dynamic strength	Same number of unweighted repetitions	4	3	Maximal voluntary contraction
Goodwill et al. (30)	RCT	7/7	7/7	Dominant leg unilateral strength training	No training	3	3	Short-interval cortical inhibition
Leung et al. (31)	RCT	24/20	33/11	Right biceps brachii training	Maintain daily activity	4	3	Maximal voluntary dynamic strength (1RM, kg), maximal load muscle action potential
Bakker et al. (32)	RCT	18/18	24/12	Single session balance board training	No training	3	3	Balance board time, short-interval cortical inhibition
Kidgell et al. (33)	RCT	15/12	18/9	Right wrist flexion maximal concentric and eccentric contractions	No training	4	3	Muscle thickness, short-interval cortical inhibition

### 3.3 Risk-of-bias assessment results

A total of 12 studies were assessed for risk of bias. Two studies were judged as having low overall risk of bias, six as having some concerns, and four as having high risk of bias. These categories describe risk-of-bias judgements rather than overall methodological quality. Most studies had relatively low risk in D1 and D4, corresponding to the randomization process and outcome measurement. In contrast, D2, D3, and D5 more frequently received Some concerns or High risk ratings, indicating concerns related to deviations from intended interventions, missing outcome data, or selective reporting (Table 2). These risk-of-bias concerns were incorporated into the interpretation of the pooled results and into the GRADE certainty assessment.

**Table 2:** Risk-of-bias assessment of included studies.

Study ID	Domains					
	D1	D2	D3	D4	D5	Overall risk of bias
Christiansen et al. (38)	Low	Low	High	Low	Low	High
Mason et al. (39)	Low	Some	Some	Low	Low	Some
Coomb et al. (40)	Low	Some	Low	Low	Some	Some
Jensen et al. (25)	Low	Some	Low	Low	Some	Some
Kidgell et al. (26)	Low	Low	Low	Low	Low	Low
Nuzzo et al. (27)	Low	Some	Low	Low	Some	Some
Siddique et al. (28)	Low	Low	Low	Low	Low	Low
Carroll et al. (29)	Low	Low	Some	Some	Low	Some
Goodwill et al. (30)	Low	Some	Some	Low	High	High
Leung et al. (31)	Low	Low	Some	Low	Some	Some
Bakker et al. (32)	Some	Low	High	Low	Low	High
Kidgell et al. (33)	Some	Some	Low	Low	High	High

D1, Randomization process; D2, Deviations from intended interventions; D3, Missing outcome data; D4, Measurement of the outcome; D5, Selection of the reported result. Low, low risk of bias; Some, some concerns; High, high risk of bias.

### 3.4 Meta-analysis results on the effect of exercise training interventions on TMS-derived neurophysiological markers in healthy individuals

#### 3.4.1 Overall effect size test for intervention effects

**Figure 2:**
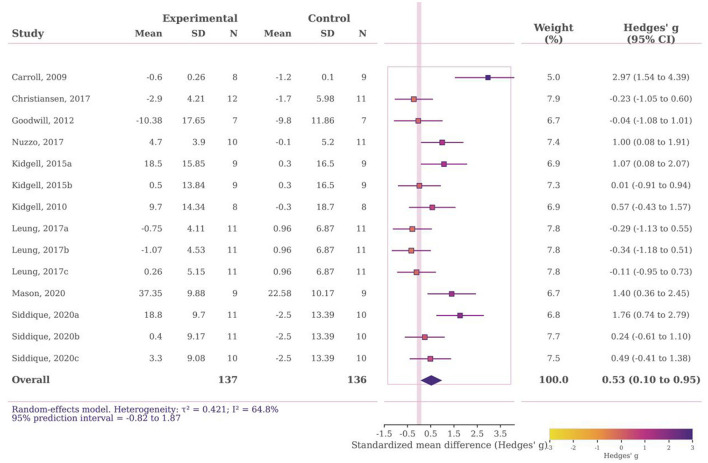
Forest plot of the effects of exercise training interventions on TMS-derived neurophysiological markers in healthy individuals.

A total of 12 randomized controlled trials (RCTs) were included. Fourteen effect sizes were available for TMS-derived neurophysiological markers, involving 137 participants in exercise groups and 136 in control groups. The heterogeneity analysis indicated moderate between-study heterogeneity (τ^2^ = 0.421, I^2^ = 64.8%); therefore, a random-effects model was applied. The pooled analysis showed a statistically significant aggregate effect of exercise training on TMS-derived neurophysiological markers (Hedges' g = 0.53, 95% CI: 0.10 to 0.95), indicating a small-to-moderate effect size (Figure 2). Because the included markers reflect different mechanisms, this result should be interpreted as a pooled TMS-marker effect rather than as evidence of uniform cortical plasticity. The 95% prediction interval ranged from −0.82 to 1.87 and crossed zero, indicating that the effect may vary across future comparable studies. These findings should therefore be interpreted cautiously.

#### 3.4.2 Exploratory subgroup analysis of modulatory variables

**Figure 3:**
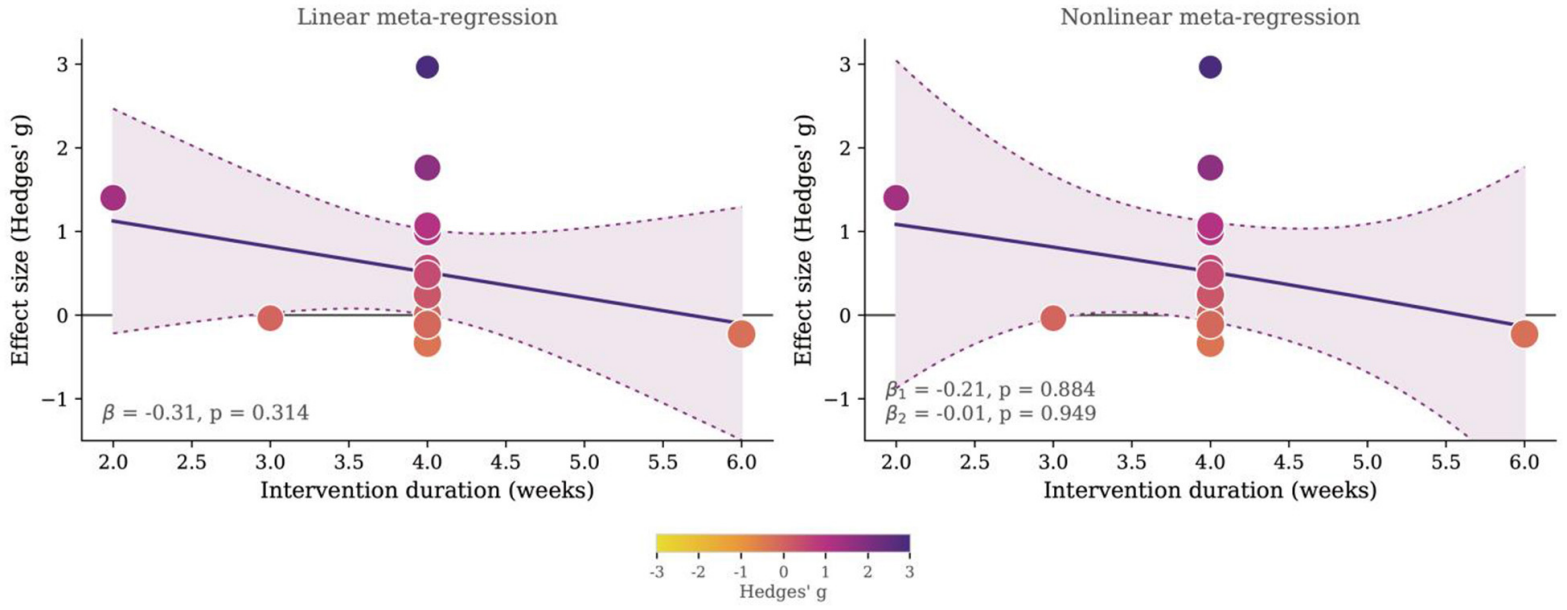
Meta-regression analysis of intervention duration for TMS-derived neurophysiological markers in healthy individuals.

Because the meta-analysis of TMS-derived neurophysiological markers included more than 10 effect sizes and showed moderate heterogeneity (I^2^ = 64.8%), exploratory moderator analyses were conducted. Intervention duration was examined using linear and nonlinear meta-regression. The linear regression coefficient for intervention duration was β = −0.31 (*p* = 0.314); in the nonlinear model, β1 = −0.21 (*p* = 0.884) and β_2_ = −0.01 (*p* = 0.949). None reached statistical significance, suggesting that intervention duration alone was not a clear source of heterogeneity (Figure 3).

Exploratory subgroup analyses are summarized in Table 3. Some within-stratum pooled estimates reached statistical significance; however, these results should not be interpreted as evidence that longer duration, higher/lower frequency, or any specific training modality is superior. The meta-regression for intervention duration was non-significant, several subgroups contained very few effect sizes (e.g., K = 1–3), and the combined-training subgroup showed high heterogeneity. These subgroup patterns should therefore be considered hypothesis-generating rather than confirmatory.

**Table 3:** Exploratory subgroup analysis for TMS-derived neurophysiological markers in healthy individuals.

Subgroup	K (participants)	Hedges' g	95% CI	*t*-value	*p*-value	I^2^	PI
Duration							
≥4 Weeks	12 (241)	0.402	[0.138, 0.667]	2.981	0.003	96%	[0.127, 0.677]
<4 Weeks	2 (32)	0.695	[−0.04, 1.43]	1.853	0.064	–	Not stable
Frequency							
≤ 3 sessions/week	13 (225)	0.402	[0.138, 0.667]	2.981	0.003	58%	[0.128, 0.676]
>3 sessions/week	1 (15)	0.695	[−0.040, 1.430]	1.853	0.064	–	Not stable
Exercise type							
Strength	10 (188)	0.425	[0.124, 0.726]	2.766	0.006	28%	[0.109, 0.741]
Skill	1 (23)	−0.225	[−1.046, 0.595]	−0.538	0.590	–	Not stable
Combined	3 (62)	0.744	[0.217, 1.271]	2.766	0.006	95%	[0.135, 1.353]

D1, Randomization process; D2, Deviations from intended interventions; D3, Missing outcome data; D4, Measurement of the outcome; D5, Selection of the reported result. Low, low risk of bias; Some, some concerns; High, high risk of bias.

### 3.5 Meta-analysis results on the impact of exercise training interventions on motor performance in healthy individuals

#### 3.5.1 Overall effect size test of intervention effect

**Figure 4:**
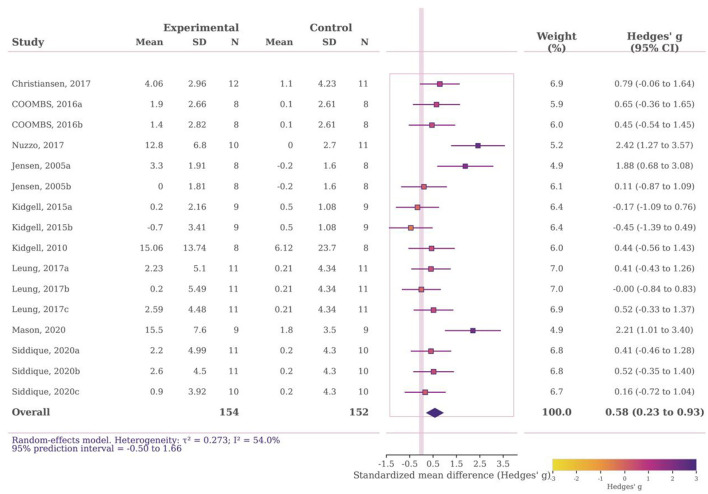
Forest plot of the effects of exercise training interventions on motor performance in healthy individuals.

A total of 12 randomized controlled trials (RCTs) were included. Sixteen effect sizes were available for motor-performance outcomes, comprising 154 participants in exercise groups and 152 in control groups. Moderate heterogeneity was observed (τ^2^ = 0.273, I^2^ = 54.0%); therefore, a random-effects model was used. The pooled effect indicated that exercise training interventions were associated with improved motor performance in healthy adults (Hedges' g = 0.58, 95% CI: 0.23 to 0.93), representing a moderate effect size (Figure 4). However, the 95% prediction interval ranged from −0.50 to 1.66 and crossed zero, suggesting that intervention effects may vary across future studies. These findings should be interpreted with caution rather than as definitive evidence of a uniform training effect.

#### 3.5.2 Exploratory subgroup analysis of modulatory variables

**Figure 5:**
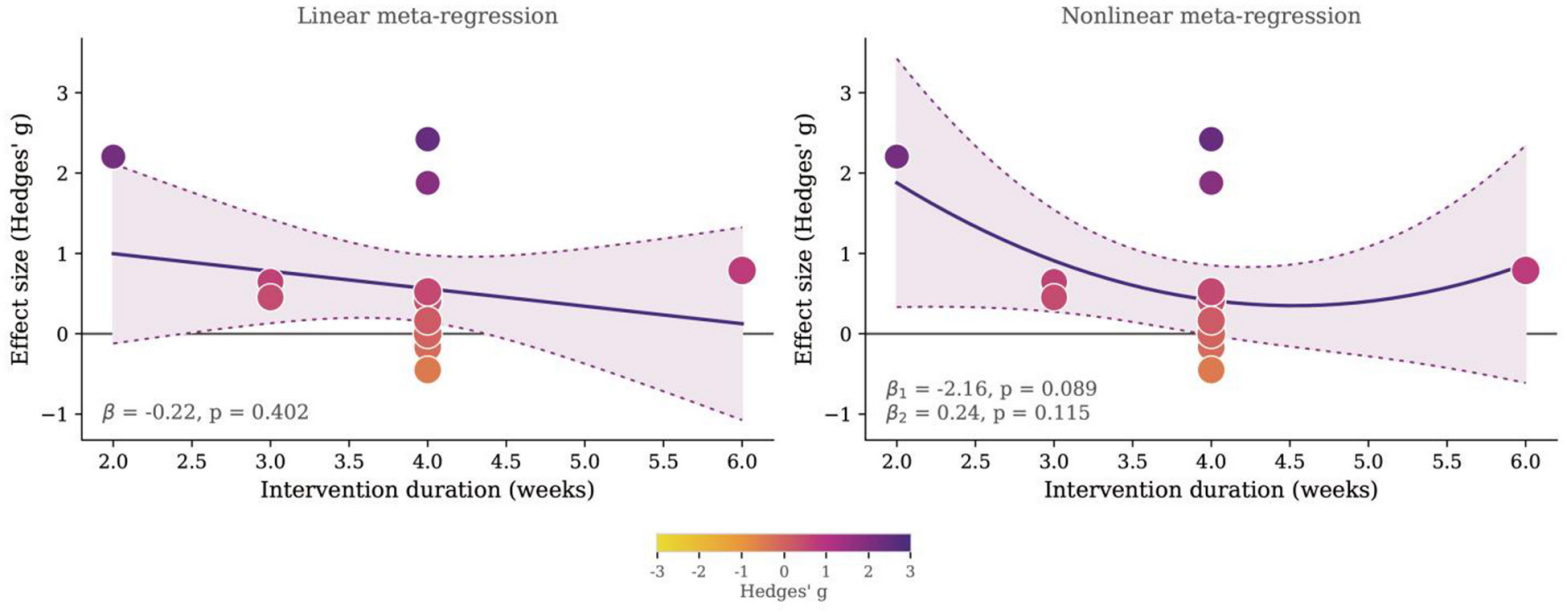
Meta-regression analysis of intervention duration for motor performance in healthy individuals.

Because the motor-performance meta-analysis included more than 10 effect sizes and showed moderate heterogeneity (I^2^ = 54.0%), exploratory moderator analyses were conducted. Intervention duration was examined as a moderator. In the linear meta-regression model, the coefficient for intervention duration was β = −0.22 (*p* = 0.402); in the nonlinear model, β1 = −2.16 (*p* = 0.089) and β_2_ = 0.24 (*p* = 0.115). None reached statistical significance, suggesting that duration alone did not explain the observed heterogeneity (Figure 5).

Exploratory subgroup analyses are summarized in Table 4. After re-checking the subgroup coding, the K values were clarified as numbers of effect sizes rather than distinct studies, and the duration and exercise-type rows were corrected so that each moderator sums to the 16 motor-performance effect sizes included in the main analysis. The resulting subgroup patterns remain exploratory. The duration meta-regression was non-significant, some subgroups were based on very small K, and several estimates had wide confidence intervals or moderate-to-high heterogeneity. Accordingly, the subgroup results should not be used as definitive evidence that one training prescription is superior.

**Table 4:** Exploratory subgroup analysis for motor performance in healthy individuals.

Subgroup	K (participants)	Hedges' g	95% CI	*t*-value	*p*-value	I^2^	PI
Duration							
≥4 weeks	13 (256)	0.45	[0.195, 0.705]	3.465	0.001	52.7%	[0.186, 0.714]
<4 weeks	3 (50)	0.99	[0.385, 1.594]	3.207	0.001	65.0%	[0.291, 1.688]
Frequency							
≤ 3 sessions/week	13 (244)	0.577	[0.312, 0.842]	4.263	<0.001	63.4%	[0.302, 0.852]
>3 sessions/week	3 (62)	0.367	[−0.136, 0.870]	1.429	0.153	0%	[−0.214, 0.948]
Exercise type							
Strength	12 (221)	0.554	[0.275, 0.833]	3.89	<0.001	66.2%	[0.264, 0.845]
Skill	1 (23)	0.788	[−0.061, 1.637]	1.819	0.069	-	Not stable
Combined	3 (62)	0.367	[−0.136, 0.870]	1.429	0.153	0%	[−0.214, 0.948]

K denotes the number of effect sizes and N denotes the summed participant count contributing to those effect sizes. For each moderator in this Table, subgroup K values sum to 16, corresponding to the total number of motor-performance effect sizes. Subgroups with very small K were interpreted as exploratory and not used for confirmatory conclusions.

### 3.6 Sensitivity analysis

**Figure 6:**
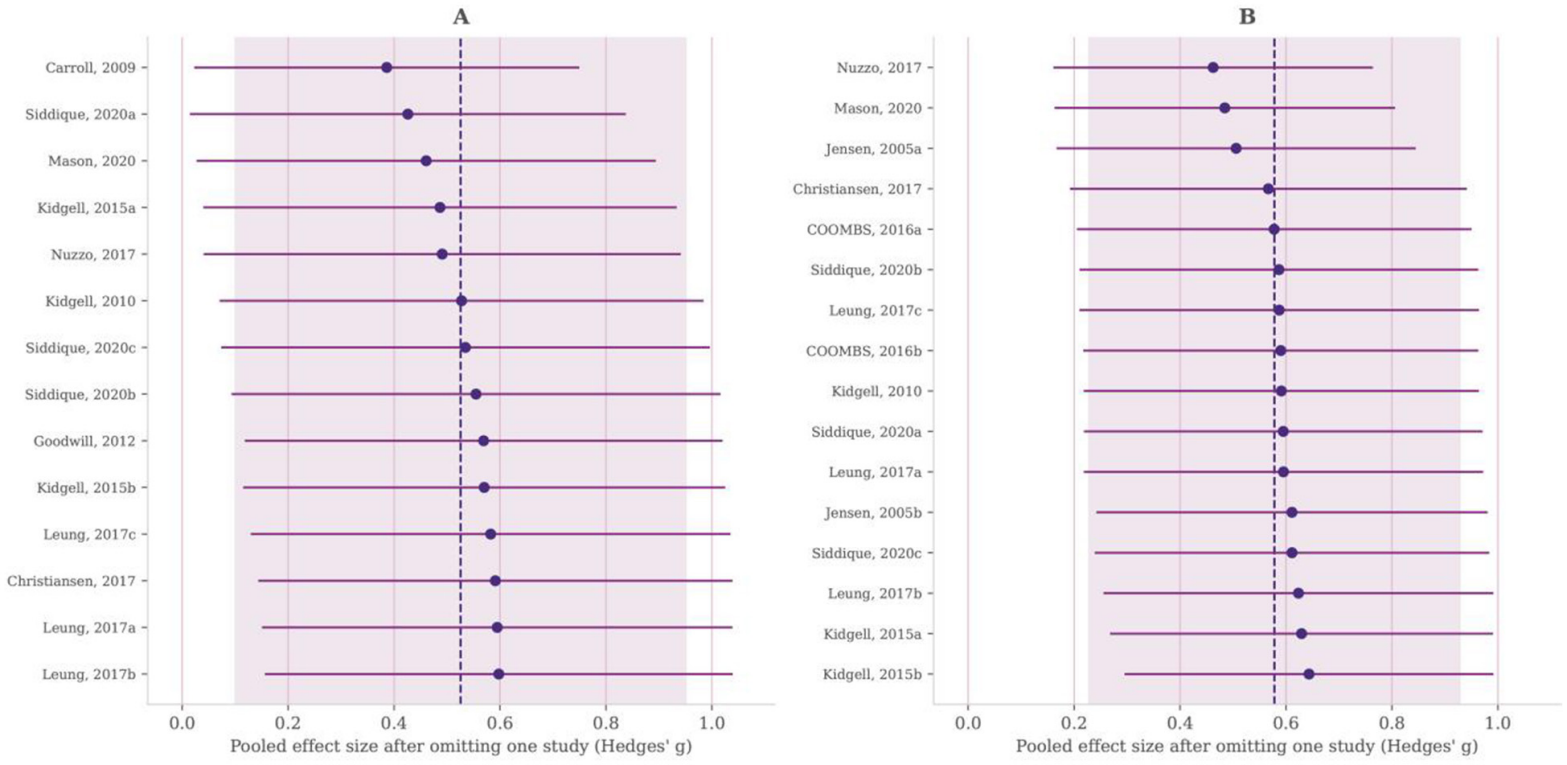
Sensitivity analysis. **(A)** Leave-one-out sensitivity analysis for TMS-derived neurophysiological markers. **(B)** Leave-one-out sensitivity analysis for motor performance.

To examine the robustness of the meta-analysis results, we conducted leave-one-out sensitivity analyses (Figure 6). The pooled effects for both TMS-derived neurophysiological markers and motor performance remained in the same direction after sequential exclusion of individual studies. This suggests that no single study fully drove the overall estimates. However, sensitivity stability does not eliminate concerns related to heterogeneity, small samples, or possible publication bias.

### 3.7 Publication bias test

**Figure 7:**
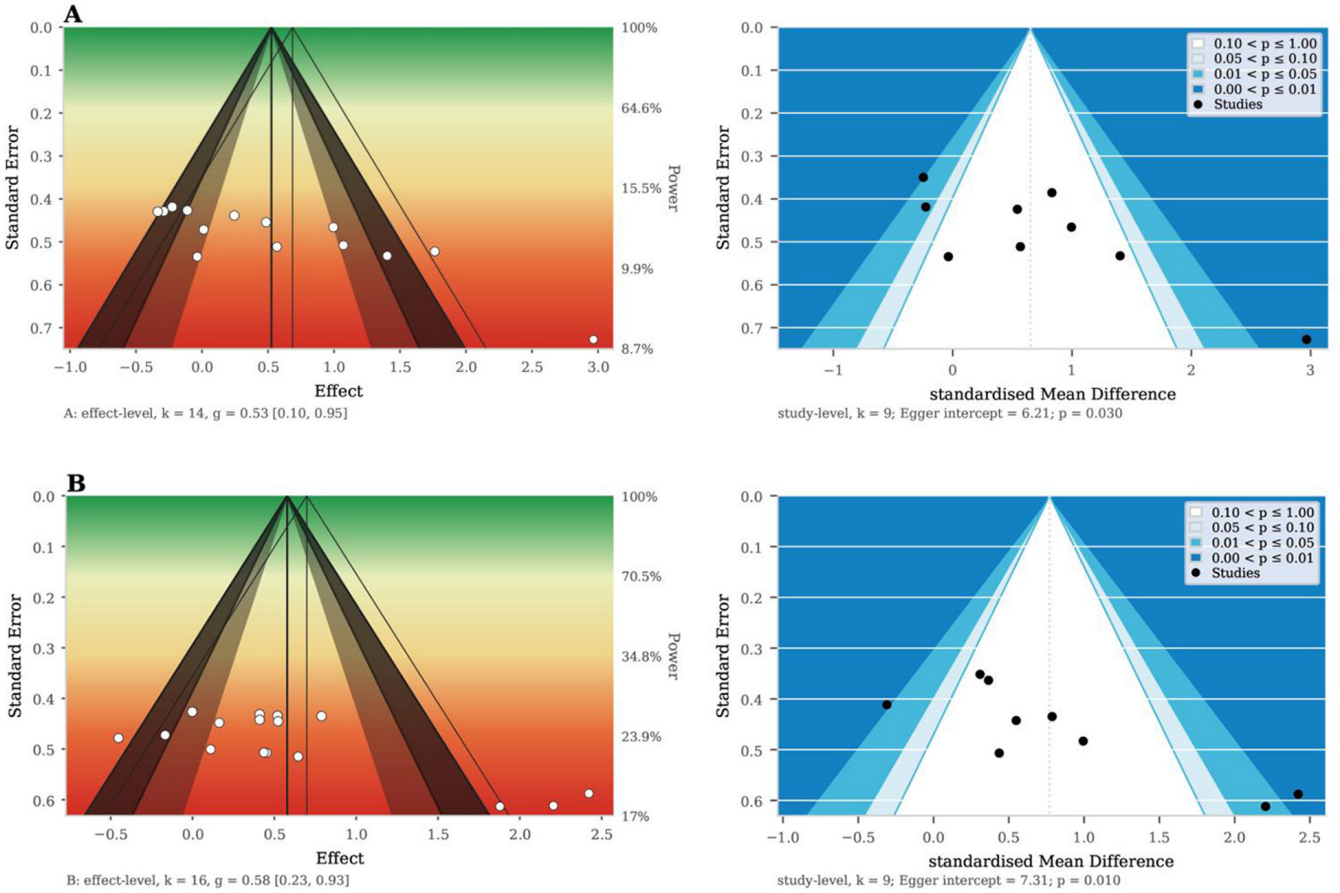
Publication bias test. **(A)** Funnel plots and contour-enhanced funnel plot for TMS-derived neurophysiological markers. **(B)** Funnel plots and contour-enhanced funnel plot for motor performance.

To assess publication bias and small-study effects, we first plotted sunset-style funnel plots based on all effect sizes. After combining multiple effect sizes from the same study into study-level independent effect sizes, contour-enhanced funnel plots and Egger regression tests were used (Figure 7). Funnel plots for both the TMS-derived neurophysiological marker outcome and the motor-performance outcome showed asymmetry. Egger tests showed intercepts of 6.21 for the TMS-derived neurophysiological marker outcome (*p* = 0.030) and 7.31 for motor performance (*p* = 0.010), suggesting possible small-study effects or publication bias. These results indicate that the pooled effects may be overestimated and should be interpreted cautiously.

### 3.8 GRADE evidence certainty

The GRADE evidence profile (Table 5) included 14 effect sizes from 12 distinct RCTs for TMS-derived neurophysiological markers and 16 effect sizes from 12 distinct RCTs for motor performance. The numbers in the GRADE table therefore refer to effect sizes contributed by the included RCTs, not to 14 or 16 separate trials. Pooled analyses indicated positive effects of exercise training on TMS-derived neurophysiological markers (Hedges' g = 0.53, 95% CI: 0.10–0.95) and motor performance (Hedges' g = 0.58, 95% CI: 0.23–0.93). The certainty of evidence for both outcomes was downgraded because of risk of bias, inconsistency, and possible publication bias. Indirectness and imprecision were not considered serious according to the prespecified GRADE criteria.

**Table 5:** GRADE evidence profile for TMS-derived neurophysiological markers and motor-performance outcomes.

Outcome	Effect sizes from studies	GRADE certainty domains				Hedges' g (95% CI)	GRADE
		Risk of bias	Inconsistency	Indirectness	Imprecision		
TMS-derived neurophysiological markers	14 effect sizes from 12 RCTs	Some serious	Some serious	Not serious	Not serious	0.53 (0.10–0.95)	⊕⊕⊕○ MODERATE
Motor Performance	16 effect sizes from 12 RCTs	Some serious	Some serious	Not serious	Not serious	0.58 (0.23–0.93)	⊕⊕⊕○ MODERATE

Explanation of columns: Outcome = main outcome category; Effect sizes from studies = number of extracted effect sizes and number of distinct RCTs contributing data. Because several RCTs contributed more than one eligible outcome, these counts are effect sizes, not separate trials. Risk of Bias, Inconsistency, Indirectness, and Imprecision are GRADE domains. Hedges' g (95% CI) = pooled standardized mean difference and confidence interval. GRADE = overall certainty of evidence.

## 4 Discussion

This systematic review and meta-analysis evaluated exercise training interventions in healthy adults using two outcome categories: TMS-derived neurophysiological markers and motor performance. The pooled effects were positive for both outcomes (Hedges' g = 0.53, 95% CI 0.10–0.95; Hedges' g = 0.58, 95% CI 0.23–0.93). These findings suggest that repeated exercise training may be associated with both central neurophysiological modulation and behavioral improvement (16–18). However, the results should be interpreted cautiously. Both outcomes showed moderate heterogeneity, and prediction intervals crossed zero. Therefore, future studies may observe effects that differ in magnitude or direction (19, 20). This interpretation is also consistent with broader background and methodological literature indicating that motor learning and exercise-related neurophysiological adaptations are task- and context-dependent, with responses varying according to activity level, learning processes, training intensity, task pacing, age, measurement approach, and complementary mechanistic evidence (41–52).

The TMS outcome should not be interpreted as a single homogeneous measure of cortical plasticity. The included studies assessed MEP amplitude, maximal MEP, motor threshold, SICI, and related markers. These measures reflect partly distinct aspects of motor cortical and corticospinal function (21–23). MEP amplitude is often interpreted as a corticospinal output measure, whereas SICI is commonly linked to intracortical inhibitory processes. For this reason, we use the term TMS-derived neurophysiological markers and interpret the pooled effect as an aggregate marker-level effect, not as evidence that exercise uniformly increases cortical plasticity.

A plausible explanation for the observed effects is that repeated, task-relevant neural activation during exercise promotes use-dependent central adaptation. Training repeatedly engages motor commands and sensory feedback from muscles, tendons, and proprioceptive systems (9, 21, 24). These processes may enhance corticospinal recruitment, alter inhibition–facilitation balance, and improve neural efficiency in a task-specific manner. Motor performance gains may then represent the behavioral expression of these neurophysiological adaptations (25–29).

The subgroup findings should be regarded as exploratory. Some strata had very small K, and several had wide confidence intervals or high heterogeneity. Although point estimates suggested possible differences by intervention duration, frequency, and modality, these patterns do not establish a stable dose–response relationship or a definitive ranking of training modalities. This is particularly important because meta-regression did not show a significant linear or non-linear association between intervention duration and effect size. The subgroup results should therefore be used to generate hypotheses for future RCTs rather than to support strong conclusions about optimal exercise prescription (25–37).

Small sample size is an important limitation of this evidence base. Many included trials had fewer than 20 participants per group, which reduces statistical power and increases the likelihood that observed effects are imprecise or exaggerated. Risk-of-bias assessment also reduced confidence: only two studies were judged to have low overall risk of bias, six had some concerns, and four had high risk. These concerns are consistent with funnel-plot asymmetry and significant Egger tests, which suggested possible small-study effects or publication bias. Although leave-one-out analyses indicated that no single study fully drove the pooled estimates, the combined effects may still be inflated by low-powered studies and selective reporting (19, 20).

## 5 Limitations

Several limitations should be acknowledged. First, the overall number of included studies was modest, and several subgroup analyses were based on very few effect sizes, reducing precision and stability. Second, many trials had small samples and were likely underpowered, increasing the risk of small-study effects and exaggerated pooled estimates. Third, substantial between-study variation was present in intervention duration, frequency, modality, target muscle groups, and outcome measures. Fourth, the neurophysiological outcome combined TMS markers with different physiological meanings, so the pooled result should not be interpreted as a single mechanism of cortical plasticity. Fifth, the analyses suggested possible publication bias, and most studies evaluated only short- to medium-term interventions. Evidence regarding long-term adaptations and retention after detraining remains limited.

### 5.1 Future directions

Future research should prioritize adequately powered, preregistered RCTs with transparent allocation, assessor blinding where possible, and complete outcome reporting. Greater standardization of intervention protocols and TMS outcomes would facilitate marker-specific meta-analyses. Future work should directly compare training modalities and test whether duration, frequency, intensity, and progression show dose–response relationships with individual neurophysiological markers and behavioral outcomes. Longitudinal studies integrating TMS, electroencephalography, electromyography, and functional neuroimaging may clarify the relationship between motor cortical/corticospinal adaptation and motor performance.

## 6 Conclusion

Exercise training interventions may be associated with improvements in motor performance and with changes in TMS-derived neurophysiological markers in healthy adults. However, the available evidence does not support strong conclusions about the superiority of specific intervention durations, frequencies, or training modalities. The heterogeneity of TMS outcomes, moderate between-study heterogeneity, prediction intervals crossing zero, small sample sizes, risk-of-bias concerns, and possible publication bias mean that the conclusions should remain cautious. Exercise training appears promising for enhancing neurophysiological and motor function, but optimal prescription parameters and mechanisms require confirmation in adequately powered, preregistered RCTs.

## Data Availability

The raw data supporting the conclusions of this article will be made available by the authors, without undue reservation.
